# Comparisons of Within-Group Instead of Between-Group Affect the Conclusions. Comment on: “Changes in Weight and Substrate Oxidation in Overweight Adults Following Isomaltulose Intake during a 12-Week Weight Loss Intervention: A Randomized, Double-Blind, Controlled Trial”. *Nutrients* 2019, *11*(10), 2367”

**DOI:** 10.3390/nu12082335

**Published:** 2020-08-05

**Authors:** Colby J. Vorland, Theodore K. Kyle, Andrew W. Brown

**Affiliations:** 1 Department of Applied Health Science, Indiana University School of Public Health-Bloomington, Bloomington, IN 47405, USA; 2 ConscienHealth, Pittsburgh, PA 15241, USA; ted.kyle@conscienhealth.org

**Keywords:** methods, differences in nominal significance, spin

We read with interest the publication by Lightowler et al., who concluded that the inclusion of isomaltulose in the context of an energy-reduced diet reduced weight and fat mass to a greater extent than sucrose [1]. We praise the authors for their use of blinding, randomization, and power calculation in their study to enhance the rigor of the experiment. Unfortunately, there are errors in the statistical conduct and interpretation that do not support these conclusions.

In the abstract, the authors write: “During the 12 weeks, both groups significantly lost weight (*p* < 0.001), which was more pronounced following [isomaltulose] (−3.2 ± 2.9 vs. −2.1 ± 2.6 kg; ***p* = 0.258**)” [1] [**emphasis added**]. The discussion also notes that “…consumption of [isomaltulose] compared to that of [sucrose] was more effective at promoting weight loss”. The authors stated in their methods that statistical significance was set at *p* < 0.05, so by the authors’ own designation, the effect on weight loss of isomaltulose was not statistically different from that of sucrose. Highlighting a beneficial effect despite a nonsignificant difference is a reporting strategy that is classified as “spin” [2].

Furthermore, throughout the paper, some conclusions about the differences between groups are made from within-group instead of between-group statistical tests, which is called a differences in nominal significance (DINS) error [3] and results in inflated type 1 error rates [4,5]. An example of this appears in the abstract: “Moreover, for participants in the [isomaltulose] group, this was accompanied by a significant reduction in fat mass ([isomaltulose]: −1.9 ± 2.5, *p* = 0.005; [sucrose]: −0.9 ± 2.6%, *p* = 0.224)” [1]. In the results, the between-group *p*-value is noted as *p* = 0.169, again not meeting the authors’ declared threshold for the appropriate between-group comparison.

Although there is much debate about whether to use a cutoff for statistical significance [6,7], the two examples discussed above do not provide strong evidence that these data are incompatible with the model under the null hypothesis of there being no difference between the groups. It is also inappropriate to solely use the differences between sample means to declare a meaningful difference between groups without accounting for the variability in those estimates [8].

Fortunately, the errors we address herein are not from the structure of the study and can therefore be easily addressed by clarifying the interpretation of the results with a corrigendum.
